# The effect of mother-infant skin-to-skin contact on Ghanaian infants’ response to the Still Face Task: Comparison between Ghanaian and Canadian mother-infant dyads

**DOI:** 10.1016/j.infbeh.2019.101367

**Published:** 2019-11

**Authors:** Frances Emily Owusu-Ansah, Ann E. Bigelow, Michelle Power

**Affiliations:** a Department of Behavioral Sciences, Kwame Nkrumah University of Science and Technology (KNUST), Ghana; b Department of Psychology, St. Francis Xavier University, Canada

**Keywords:** Ghanaian mother-infant dyads, Skin-to-skin contact, Still Face Task

## Abstract

The effect of mother-infant skin-to-skin contact on Ghanaian infants’ developing social expectations for maternal behavior was investigated. Infants with high and low mother-infant skin-to-skin contact experience in the infants’ first month engaged with their mothers in a Still Face Task at 6 weeks of age. Infants with high skin-to-skin contact experience, but not those with low skin-to-skin contact experience, demonstrated the still face effect with their smiles. Infants with both high and low skin-to-skin contact experience demonstrated the still face effect with their visual attention. The behaviors of the Ghanaian infants and their mothers during the task were compared to archival evidence of Canadian mother-infant dyads’ behaviors in skin-to-skin and control groups who engaged in the Still Face Task at the infant ages of 1 and 2 months. Similarities and differences between the behaviors of the mother-infant dyads in the two cultures were assessed.

## Highlights

• Ghanaian mother-infant dyads engage in skin-to-skin contact in infants’ first month.
• Infants show the still face effect with attention at 6 weeks.
• Only infants with high skin-to-skin contact show the still face effect with smiles.
• Similarities and differences to Canadian mother-infant interactions are compared.

## 1 Introduction

Developmental psychologists increasingly are exploring how infants’ biological maturation intersects with parenting cultural goals and styles of interaction. Parents from different cultural contexts value different parenting goals that affect parenting interactive practices, which in turn shape infants’ behavior (Kärtner et al., 2008; Keller, 2007; Keller et al., 2004). Distal parenting practices, focusing on face-to-face contexts and object play, are prevalent in Western industrialized societies where parents tend to socialize their infants toward goals of independence and autonomy. Proximal parenting practices, focusing on physical contact and body stimulation, are prevalent in many non-Western societies where parents socialize their infants toward goals of interdependence and relatedness with family and community. Although this duality is likely an oversimplification of parenting goals and practices as complex cultural cross-overs due to globalization as well as individual differences exist in any culture, it is clear that the way of engaging infants prevalent in Western societies is not universal. Yet infants’ biological maturation is universal. Infants are biologically predisposed to engage with others. Their biological predispositions interact with parenting practices early in life and adapt to cultural demands.

Mother-infant skin-to-skin contact (SSC) is a method of caring for young infants. The infant is placed between the mother’s breasts, dressed only in a diaper, so that frontal body contact of mother and infant is skin-to-skin.

SSC benefits newborns’ neuro-physical adjustment. By engaging in SSC with her infant, the mother provides warmth and stimulation that is believed to simulate the prenatal environment (Whitelaw & Sleath, 1985). Across a number of cultures, SSC for infants early in life has been shown to stabilize infant temperatures, heart rates, respiratory rates, gastrointestinal adaptations (Bauer, Sontheimer, Fischer, & Linderkamp, 1996; Bergman, Linley, & Fawcus, 2004; Bystrova et al., 2003; Charpak, Ruiz-Pelaez, Figueroa de Calume, & Charpak, 1997; Cleary, Spinner, Gibson, & Greenspan, 1997; Conde-Agudelo, Diaz-Rossello, & Belizen, 2000; Feldman, Eidelman, Sirota, & Weller, 2002; Fohe, Kropf, & Avenarius, 2000; Ludington-Hoe, Anderson, Swinth, Thompson, & Hadeed, 2004; Moore, Anderson, & Bergman, 2007; Mori, Khanna, Pledge, & Nakayama, 2010), and reduce pain from routine medical procedures (Gray, Miller, Philipp, & Blass, 2002; Gray, Watt, & Blass, 2000).

Studies investigating the effects of SSC beyond the newborn period have shown that the effects extend to infants’ social cognitive development. Feldman and colleagues (Feldman, Eidelman, et al., 2002; Feldman, Weller, Sirota, & Eidelman, 2002) found that, compared to infants who did not receive SSC, infants at 3 months who received SSC as newborns had more interest in novel stimuli, and at 6 months were more advanced in toy exploration and shared attention with mother. Ohgi et al. (2002) found that infants at 6 months who had previous SSC showed better orientation to social and non-social objects. At 12 months, infants with SSC experience performed better on infant development scales than infants without such experience (Feldman, Eidelman, et al., 2002; Ohgi et al., 2002; Tessier et al., 1998).

Multiple reasons have been proposed for why SSC early in life would facilitate infants’ subsequent social cognitive development (Feldman, Eidelman, et al., 2002; Feldman, Weller, et al., 2002). The post-birth period constitutes a sensitive period for maternal contact in animal and human models (Field, 1995; Lehmann, Stohr, & Feldon, 2000; Scafidi et al., 1990; Wigger & Neumann, 1999), and tactile and proprioceptive stimulation are especially important during this time (Gottlieb, 1976, Gottlieb, 1991). SSC improves infants’ state organization, particularly infants’ sleep/wake cycles, as well as improves stress reactivity and physical maturation (Feldman, Weller, et al., 2002; Michelsson, Christensson, Rothganger, & Winberg, 1996). Such early physiological and behavioral regulation predicts later cognitive development (Beckwith & Parmelee, 1986; Doussard-Roosevelt, Porges, Scanlon, Alemi, & Scanlon, 1997; Feldman, Greenbaum, Yirmiya, & Mayes, 1996; Sigman, Cohen, & Beckwith, 1997). The close proximity between mother and infant in SSC also facilitates infants’ ability to recognize and respond to their mothers and mothers’ ability to recognize and become familiar with their infants’ signals (Feldman, 2004). Mothers who are sensitive to their young infants’ signals engage in more frequent and positive mother-infant interactions. Mothers who provide SSC for their infants report more positive maternal feelings, positive perceptions of their infants, less depression, and more empowerment in their parenting role (Affonso, Bosque, Wahlberg, & Brady, 1993; Johnson, 2007; Neu, 1999; Roller, 2005; Tessier et al., 1998; Whitelaw, 1990). Although most studies of the effects of SSC on maternal feelings and behavior have been conducted only in the newborn period, some have shown increased maternal sensitivity and maternal behaviors of holding, touch, and infant-directed speech throughout the infants’ first year (Bigelow, Littlejohn, Bergman, & McDonald, 2010; DeChateau and Wiberg, 1977, DeChateau and Wiberg, 1984; Feldman, Eidelman, et al., 2002). SSC promotes infants’ social cognitive development by affecting infants’ behavioral and physiological regulation and mothers’ maternal behaviors (Feldman, 2004). Thus in mother-infant interactions, infants with SSC experience may have enhanced understanding of the relation between their own actions and their mothers’ social responsiveness to them.

The Still Face Task has been used to examine infants’ awareness of others’ social behavior toward them. This task, first reported in a seminal study by Tronick and colleagues (Tronick, Als, Adamson, Wise, & Brazelton, 1978), has mothers or other social partners engage infants in normal face-to-face interaction, then become suddenly still and expressionless, and then resume normal interaction. Infants tend to exhibit reduced visual attention and decreased positive affect, as demonstrated by changes in smiling and non-distress vocalizations, during the still face phase compared to the interactive phases. Such changes in the infants’ behavior, known as the still face effect, have been shown in numerous studies, typically with infants between 2 and 9 months of age (but see Bigelow & Power, 2012, below for effects in younger infants) and are found regardless of procedural variations, such as length of the episodes (Adamson & Frick, 2003; Mesman, van IJzendoorn, & Bakermans-Kranenburg, 2009).

Bigelow and Power (2012) conducted a longitudinal quasi-experiment with Canadian infants in SSC and control groups in which infants engaged with their mothers in the Still Face Task at ages 1 week, 1 month, 2 months, and 3 months. At 1 week, infants in both groups demonstrated the still face effect with their visual attention that continued to be demonstrated at each of the following ages, thus showing that even in the newborn period infants can detect changes in their mothers’ interactive behavior. At 1 month, differences between the groups appeared. Infants in the SSC group began responding to changes in their mothers’ behavior with their affect, suggesting they were responding to violations of their expectations for affect sharing. Infants in the control group did not show affect changes to the phases of the Still Face Task until 2 months. At 3 months, infants in the SSC group increased their non-distress vocalizations in the still face phase, indicative of social bidding to their non-responsive mothers, whereas infants in the control group decreased their non-distress vocalizations in the still face phase. SSC accelerated infants’ social expectations for their mothers’ behavior and enhanced infants’ awareness of themselves as active agents in eliciting social interactions with their mothers.

A focus of the present study was to investigate whether SSC would increase infants’ early expectations for the social behaviors of others in a culture with proximal parenting practices. Although SSC studies have been conducted in various cultural contexts, the focus in these contexts has primarily been on the effects of SSC on infants’ neuro-physical adjustments shortly after birth (see Mori et al., 2010, for meta-analysis including SSC studies from 18 different countries). The findings from these studies show similar benefits of SSC to newborns’ physiological adaptations to postnatal life. However, the effect of SSC on infants’ social expectations of others in cultures with different parenting goals and practices has been less well researched.

Ghana has a culture with proximal parenting practices. As in many African countries, Ghanaian mothers carry their infants by wrapping them onto their backs as they go about their daily chores or business. Infants sleep in the same bed with their mothers and spend most of their day with their mothers, primarily wrapped on their mothers’ backs. Frontal SSC is not the norm, yet mother-infant tactile contact is high compared to mother-infant dyads in Western societies (Kärtner et al., 2008; Keller et al., 2004).

Ghanaian parenting goals and practices resemble those found in other West African societies, of which the Nso of the Cameroon have been the most thoroughly studied. Kärtner and colleagues (Kärtner et al., 2008; Kärtner, Keller, & Yovsi, 2010) compared Nso and German mother-infant interactions. They found that Nso mothers were more directive in their maternal interactions and had more physical contact with their infants. Nso infants experienced less face-to-face interactions and reduced smiling and verbal turn-taking with their mothers. Yet Nso infants experienced enhanced physical closeness with their mothers due to being carried on their mother’s body and sleeping with her. Such practices by Nso mothers facilitate proximal parenting goals of interdependence, such as obedience, deference, and collective responsibility. Maternal responsiveness to their infants was similar across the two cultures, albeit manifested differently. German mothers were more visually responsive with gaze, smiles, and facial expressions; Nso mothers were more tactually responsive with touch and physical stimulation (Kärtner et al., 2008, Kärtner et al., 2010). These cultural differences emerged between the infants’ second and third month. Mothers in both cultures responded readily to infants’ non-distress vocal signals. For German mothers, maternal verbal responses are the norm; for Nso mothers, physical contact responses are prevalent. Mother-infant verbal turn-taking may be less necessary when physical closeness, such as being carried on the mother’s body, allows for ready physical responsiveness (Mesman et al., 2018).

Mother-infant interaction is a bidirectional process in which each partner affects the other. Although this process begins at birth, it increases around 2 months. This 2 month transition is thought to be due to infants’ neural maturational processes and their experience with social partners who reinforce and contingently respond to the infants’ social behaviors (Wörmann, Holodynski, Kärtner, & Keller, 2012). Around 2 months of age, infants’ maturational processes allow them to become more alert, to maintain posture and attention for longer periods, and to systematically explore the internal features of the face (Haith, Bergman, & Moore, 1977; Hopkins et al., 1990; Wolff, 1987). Infants become more aware and interested in social partners at this time (Rochat, 2001). Infants’ visual attention, smiling, and non-distress vocalizations increase (Spitz, 1965; Trevarthen, 1979; Wolff, 1987). They become more responsive (Henning, Striano, & Lieven, 2005) and show effortful patterns of communication (Lavelli and Fogel, 2002, Lavelli and Fogel, 2005).

Kärtner et al. (2010) found that Nso infants’ alertness increased between 6 and 8 weeks, showing evidence of neural maturation associated with the 2 month transition. The culturally specific differences between German and Nso mothers’ responses to their infants occurred around this time. These cultural differences were due primarily to changes in the way German mothers responded to their infants. Between infants’ second and third months, German mothers reduced their tactile responses and increased their gaze and facial responses to their infants, whereas Nso mothers continued to respond to their infants with high levels of tactile responsiveness and did not increase their visual responses. Nso mother-infant dyads engaged in mutual gaze less often than German mother-infant dyads; nevertheless, when in face-to-face interaction, Nso mothers smiled as much as German mothers and Nso infants at 6 weeks gazed and smiled at their mothers as much as German infants at this age (Kärtner et al., 2010; Wörmann et al., 2012).

### 1.1 The present study

Infants’ response to the Still Face Task appears to be robust in that it is found in infants of varying ages and in normative and at-risk samples, yet very few studies have conducted the task with infants in non-Western cultures (Mesman et al., 2009). To date only three such studies have been published: Kisilevsky et al. (1998) tested 3 to 6 month old infants in China, Hsu and Jeng (2008) tested 2 month old infants in Taiwan, and Yato et al. (2008) tested 4 and 9 month old infants in Japan. Infants in these studies responded to the task with the still face effect, similar to infants in Western cultures. Yet participants in these studies were from urban, predominantly highly educated, middle- to high-income families, where mother-infant interactive practices may be affected by those of Western cultures. Education, with accompanying changes in income and living standards, influences parenting goals toward independence and autonomy (Kağitçibaşi, 1996; LeVine, Miller, Richman, & LeVine, 1996) as well as increases exposure to Western parenting practices through international acquaintances and travel. To date, no study has used the Still Face Task in an African culture where proximal parenting practices are prevalent. Nevertheless, infants as they approach the 2 month transition may be responsive to the changes in their mothers’ behavior during the task and SSC may accelerate infants’ affective responses to the task, as it did in the Bigelow and Power (2012) study.

The present study examined the effect of SSC experience on the behavior of Ghanaian mother-infant dyads in the Still Face Task at the infant age of 6 weeks, when the infants were on the cusp of the 2 month transition. Although at prenatal checkups women were informed about SSC, the amount the Ghanaian mothers provided for their infants varied. The dyads were subsequently divided based on a median split (Bigelow & Power, 2014) into those with high and low SSC experience. Their behavior was compared to archival data from mother-infant dyads in the Bigelow and Power (2012) study at the infant ages of 1 and 2 months. Hypotheses were four: (1) Ghanaian infants with both high and low SSC experience would show the still face effect with their visual attention, like the Canadian infants at both 1 and 2 months; (2) Ghanaian infants with high SSC experience, but not those with low SSC experience, would show the still face effect with their affect, similar to the Canadian infants at 1 month; (3) Ghanaian infants would show overall behaviors of visual attention, smiling, non-distress vocalizations, frowning, and distress vocalizations that are intermediate between those behaviors found in the Canadian infants at 1 and 2 months; and (4) Ghanaian mothers would show more tactile and less verbal behaviors but similar visual attention and smiling behaviors during the interactive phases of the Still Face Task compared to the Canadian mothers.

## 2 Method

### 2.1 Participants

#### 2.1.1 Ghanaian sample

Mothers were recruited prior to the birth of their infants during prenatal visits at the Komfo Anokye Teaching Hospital, a large public hospital in Kumasi, the capital city of the Ashanti Region and the second largest city in Ghana. The Antenatal Clinic at Komfo Anokye Teaching Hospital serves pregnant women residing in the urban area and surrounding villages. The Ashanti Region adheres to a matrilineal tradition in which people have an attachment to villages of their mothers’ family. Pregnant women typically spend time in these villages prior to, and after, the birth of their infants. Thus traditional means of child care are prevalent for both urban and village women. Although English is the official language of Ghana, in the Ashanti Region Twi is the common language. All the communications with the mothers were conducted in Twi by native speakers.

The participants were 26 infants (14 males) and their mothers. The mean age of the mothers at the infants’ birth was 28.1 years (*SD* = 4.0 years). The percentage of mothers with a university degree was 15%, 15% had some post-secondary training, 31% had only a high school diploma, and 39% were without a high school diploma. The mothers were predominantly from the ethnic group Akan (92%) with a minority from Ewe (8%). For 40% of the mothers, this was their first child; 28% of the mothers had one previous child, and 32% of the mothers had two or more previous children. All the mothers had telephones, necessary for SSC reporting. The infants’ mean gestation age was 38.3 weeks (*SD* = 1.9 weeks). Their mean birth weight was 3005.3 g (*SD* = 489.3 g). The videotaped mother-infant Still Face Task was conducted at the infants’ 6 week checkup (*M* age = 46 days, *SD* = 5 days).

#### 2.1.2 Canadian sample

The participants were 80 infants (38 males) and their mothers. Mothers were recruited prior to the birth of their infants through perinatal clinics at two hospitals with similar demographics in northeastern Canada. One hospital recruited for the SSC group and one hospital recruited for the control group. Approximately halfway through the study, the recruitment sites were switched; the former SSC site became the control site and vice versa. Dyads of the recruited mothers were included if the infants were over 37 weeks gestation age and had no medical problems. Twenty-eight dyads were in the SSC group and 52 dyads were in the control group. Socioeconomic status (SES) of the infants’ families was measured by a Canadian index (Blishen, Carroll, & Moore, 1987) based on education and income. In the index, occupations are divided into 514 groups, ranging in SES scores of 17.81–101.75 (*M* = 42.74, *SD* = 13.28). The scores of the higher status parent in the infants’ families yielded a SES mean score of 50.41 (*SD* = 11.80). The mean age of the mothers at the infants’ birth was 29.7 years (*SD* = 5.0 years). The percentage of mothers with a university degree was 42%, 41% had some university education, 16% had only a high school diploma, and 1% were without a high school diploma. The racial-ethnic composition of the mothers was 99% non-Hispanic White and 1% Asian. For 47% of the mothers, this was their first child, 29% of the mothers had one previous child, and 24% of the mothers had two or more previous children. The infants’ mean birth weight was 3647.11 g (*SD* = 530.95 g). The videotaped mother-infant Still Face Tasks used for comparison with the Ghanaian mother-infant dyads were conducted when the infants were 1 month (*M* = 32 days, *SD* = 5 days) and 2 months (*M* = 64 days, *SD* = 8 days).

### 2.2 Procedure

#### 2.2.1 In Ghana

The study received ethical clearance from the first author’s university research ethics board. Women coming for prenatal checkups at Komfo Anokye Teaching Hospital were told about SSC, encouraged to provide SSC for their infants, and asked if they would be willing to participate in the study, which involved keeping daily records of the amount of SSC they provided for their infants through the infants’ first month and engaging in a videotaped Still Face Task with their infants at the 6 week checkup. Women who agreed to participate filled out demographic forms. The first author was notified when the participating mothers gave birth and was given access to the infants’ sex, birth weight, and gestation age. A research assistant telephoned participating mothers weekly through the infants’ first month to gather the daily records of amount of SSC the mothers provided during the previous week. At the infants’ 6 week checkup, the mother and infant engaged in the Still Face Task in a private room in the hospital.

In the Still Face Task, the mother and infant sat facing each other approximately 50 to 60 cm apart. The infant sat in an infant car seat that was situated on a table. The mother sat in a chair that allowed her to be at eye level with the infant. Behind and to the side of the infant was an upright mirror (60 cm × 40 cm) in a frame that could be angled to reflect the mother seated opposite the infant. The angle was typically 70–75°. The research assistant videotaping the Still Face Task was behind and to the side of the mother, out of the direct view of the mother and infant. The videotape recorded the infant (full frontal body) and the mother’s reflection (frontal body from the waist up).

The Still Face Task consisted of three phases that sequentially followed each other without pause: initial interactive phase, still face phase, reunion phase. For the initial interactive phase, the mother was asked to interact with her infant as she wished for two minutes. For the still face phase, she was asked to become still with a neutral expression, looking at her infant but not talking or touching the infant for one minute. For the reunion phase, the mother was asked to interact with her infant again as she wished for two minutes. The research assistant gave the mother a verbal cue at the beginning of each phase and at the end of the task.

The behavior of the mother and infant was scored in the lab of the second author on the Observer Video-Pro 5.0 (Noldus Information Technology, 2003) computer software program by a coder blind to the amount of SSC mothers provided. Infants were scored for duration of visual attention, and positive and negative affect in facial expressions (smiles, frowns) and in vocalizations (non-distress vocalizations, distress vocalizations) during each of the three phases of the Still Face Task. Visual attention was scored as the presence or absence of looking at the mother’s face. Smiles were scored as upward lip movements with or without vocalizations. Frowns were downward lip movements with or without vocalizations. Non-distress vocalizations excluded distress vocalizations (fussing, crying) and digestive sounds (e.g., burps, hiccups). Distress vocalizations excluded non-distress vocalizations and digestive sounds. The duration times were converted to percentage of time within each phase. Mothers were scored for duration of visual attention to the infant’s face, smiles, vocalizations, and physical contact with their infants during the two interactive phases of the task. Mothers’ vocalizations, which were all non-distress vocalizations, were further coded as arousing (energetic, highly stimulating) or neutral (excluding arousing vocalizations). Mothers’ physical contact with their infants was coded as arousing (e.g., pumping the infant’s legs), attention getting (e.g., tapping the infant’s face when not looking at mother), soothing (e.g., gentle stroking), adjusting (e.g., repositioning infant), or passive holding (e.g., hands passively on infant’s body). The duration times were converted to percentage of time within the interactive phases. The coder was blind to the amount of SSC the mothers provided. Coding of the infant and mother behaviors followed that of previous studies (e.g., Bigelow, 1998; Bigelow & Power, 2012; Bigelow & Walden, 2009), with the addition of mothers’ type of vocalizations (arousing, neutral) and physical contact (arousing, attention getting, soothing, adjusting, passive holding).

For reliability purposes, a second coder, who was also blind to the amount of SSC mothers provided, independently scored the behaviors of 19% of the dyads. For infant and mother behaviors, the range of intraclass correlations, absolute type with raters random, was between .821 and .997 (all *p* ≤ .01).

#### 2.2.2 In Canada

After the study received ethical clearance from the two participating hospitals and the second author’s university research ethics board, the perinatal clinics in the two hospitals distributed Consent to be Contacted Forms to pregnant women in the third trimester of their pregnancy. The women who signed the form were contacted by a research assistant, who explained the study. Mothers who agreed to participate had notices put on their medical charts so that attending nurses would notify the research assistants when the women gave birth. Research assistants (*N* = 8) visited the mother-infant dyads in their homes when the infants were 1 week, 1 month, 2 months, and 3 months. Mothers were seen by the same research assistant from the contact interview through the data collection visits.

Mothers in the SSC group were requested to provide six hours of SSC with their infants cumulative throughout the day during the infants’ first week, and then two hours per day until the infants were one month. No request for mother-infant SSC was made to control group mothers. Mothers in both the SSC and control groups recorded the amount of SSC they provided to their infants each day and records were collected at each visit.

The present report presents the results from the Still Face Task conducted in the home when the infants were 1 month and 2 months of age. The setup and procedure of the Still Face Task was identical to that done in Ghana with the exception that the initial interactive phase lasted for 3 min.

Coding of the mothers and infants was done in the lab of the second author as described in the Ghana sample by a coder blind to whether the dyads were in the SSC or control groups. For reliability purposes, a second coder, who was also blind to whether the dyads were in the SSC or control group, independently scored the behaviors of 13% of the dyads. For infant and mother behaviors, the range of intraclass correlations, absolute type with raters random, was between .824 and .999 (all *p* ≤ .01).

## 3 Results

### 3.1 Plan of analysis

In preliminary analyses, analyses of variance (ANOVAs) were conducted on the demographics of the Ghanaian mothers and infants in the high and low SSC groups and on the demographics of the Canadian mothers and infants in the SSC and control groups. ANOVAs also compared the amount of SSC the Ghanaian mothers in the high and low SSC groups did in their infants’ first week and in their infants’ weeks 2 through 4 with the amount of SSC the Canadian mothers in the SSC and control groups did in their infants’ first week and in their infants’ weeks 2 through 4.

To test Hypotheses 1 and 2, mixed ANOVAs with the repeated variable phase (initial interactive, still face, reunion) and the between variable SSC group (high, low) were conducted on Ghanaian infants’ visual attention (Hypothesis 1) and smiles, non-distress vocalizations, frowns, and distress vocalizations (Hypothesis 2) during the Still Face Task. Significant group x phase interactions were followed by *t-*test pairwise comparisons. Similar ANOVAs (between variable group: SSC, control) and follow-up comparisons were conducted on the Canadian infants’ behaviors at 1 month and at 2 months. To test Hypothesis 3, ANOVAs with the between variable country (Ghana, Canada) compared the overall infant behaviors during the Still Face Task of Ghanaian infants (visual attention, smiles, non-distress vocalizations, frowns, distress vocalizations) with those behaviors of Canadian infants at 1 month and at 2 months. To test Hypothesis 4, ANOVAs with the between variable country (Ghana, Canada) compared the Ghanaian mothers’ behaviors toward their infants (visual attention, smiles, vocalizations, physical contact) during the interactive phases of the task with these behaviors of the Canadian mothers when their infants were 1 month and 2 months. Follow-up ANOVAs (between variable country: Ghana, Canada) compared the Ghanaian mothers’ type of vocalizations (arousing, neutral) and type of physical contact with their infants (arousing, attention getting, soothing, adjusting, passive holding) with those of the Canadian mothers when their infants were 1 month and 2 months.

### 3.2 Preliminary analyses

Dyads in the Ghanaian sample were divided into high and low SSC groups based on a median split of the amount of SSC reported during the infants’ first month. ANOVAs conducted on the demographics of the mothers and infants indicated there was no significant difference between the mothers in the high and low SSC groups in maternal age, education, or number of previous births; or in their infants’ sex, gestation age, or birth weight.

For the dyads in the Canadian sample, ANOVAs conducted on the demographics of the mothers in the SSC and control groups indicated there were no significant differences between the groups in maternal education, number of previous births, or SES, although the mothers in the SSC group were slightly older (*M* = 32.0 years, *SD =* 5.5 years) than the mothers in the control group (*M =* 28.4 years, *SD =* 4.1 years). Infants in the SSC and control groups did not differ in sex, gestation age, or birth weight.

Table 1 shows the amount of SSC the Ghanaian mothers in the high SSC and low SSC groups and the Canadian mothers in the SSC and control groups provided for their infants in the infants’ first week and in the weeks 2 through 4. There was no significant difference in the amount of SSC provided by Ghanaian and Canadian mothers in their infants’ first week either in the Ghanaian high SSC group and the Canadian SSC group or in the Ghanaian low SSC group and the Canadian control group. However, in the infants’ weeks 2 through 4, Ghanaian mothers provided more SSC in the high SSC group than Canadian mothers provided in the SSC group, *F* (1, 38) = 31.29, *p* < .001, η_p_^2^ = .452, and Ghanaian mothers in the low SSC group provided more SSC than the Canadian mothers did in the control group, *F* (1, 62) = 58.54, *p* < .001, η_p_^2^ = .486, but less SSC than Canadian mothers did in the SSC group, *F* (1, 38) = 16.44, *p* < .001, η_p_^2^ = .302.

**Table 1:** Mean hours per day of mother-infant skin-to-skin contact during the infants’ first week and during weeks 2 through 4.

Infant Age		
Group	Week 1	Weeks 2 through 4
Ghana		
High Skin-to-Skin	3.9 (2.2)	5.2 (1.7)
Low Skin-to-Skin	0.8 (0.6)	1.5 (0.9)
Canada		
Skin-to-Skin	5.0 (1.4)	2.8 (1.0)
Control	0.5 (0.9)	0.2 (0.4)

*Note.* Standard deviations are in parentheses.

**Fig. 1:**
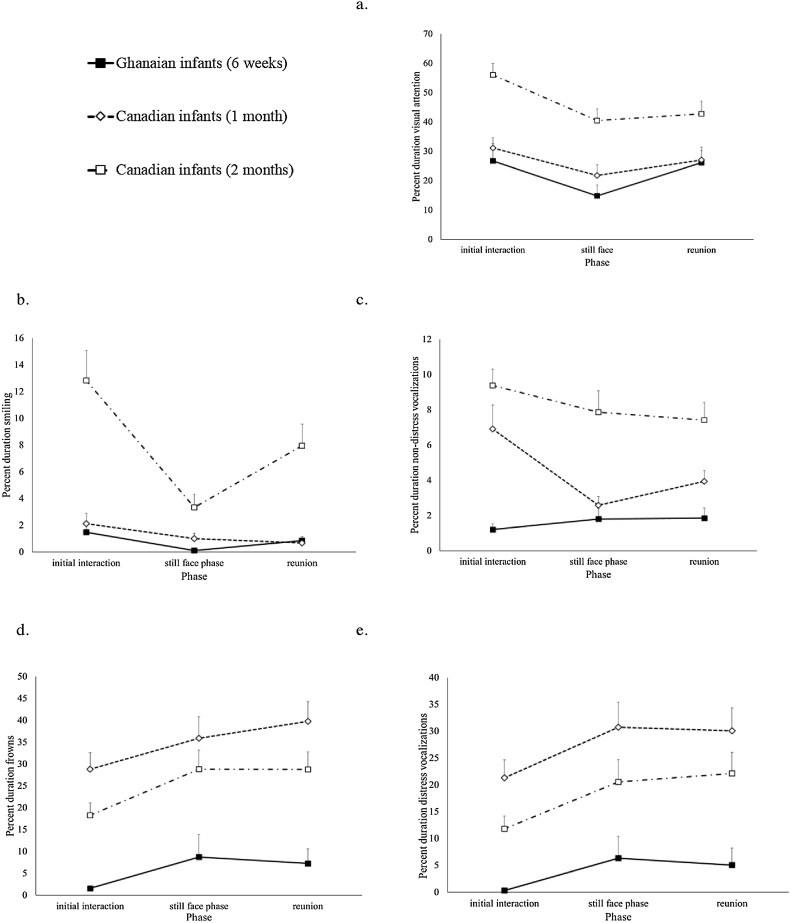
Infant behaviors across the phases of the Still Face Task for Ghanaian infants at 6 weeks and Canadian infants at 1 and 2 months: (a) visual attention, (b) smiling, (c) non-distress vocalizations, (d) frowning, (e) distress vocalizations. Vertical bars represent standard errors.

Fig. 1 shows infants’ behaviors (visual attention, smiling, non-distress vocalizations, frowning, distress vocalizations) across the phases of the Still Face Task for the Ghanaian infants at 6 weeks and the Canadian infants at 1 and 2 months.

### 3.3 Hypothesis 1: Ghanaian infants with both high and low SSC experience would show the still face effect with their visual attention, like the Canadian infants at 1 and 2 months

Mixed ANOVAs with the repeated variable phase (initial interactive, still face, reunion) and between variable SSC group (high, low) conducted on the Ghanaian infants’ duration of visual attention showed a significant main effect for phase, *F* (2, 48) = 5.05, *p* = .010, η_p_^2^ = .174, indicating the still face effect, with no group main effect or interaction effect. The Canadian infants showed similar results from mixed ANOVAs with the repeated variable phase (initial interactive, still face, reunion) and the between variable group (SSC, control) conducted on the infants’ visual attention at 1 and 2 months. At 1 month, the Canadian infants’ visual attention showed a significant main effect for phase, *F* (2, 152) = 4.65, *p* = .011, η_p_^2^ = .058, with no group main effect or interaction effect. These results were replicated at 2 months (phase main effect: *F* (2, 146) = 18.34, *p* < .001, η_p_^2^ = .201). As can be seen in Fig. 1a, the Ghanaian infants, like the Canadian infants at 1 and 2 months, discriminated between the phases of the Still Face Task with their visual attention.

### 3.4 Hypothesis 2: Ghanaian infants with high SSC experience, but not those with low SSC experience, would show the still face effect with their affect, similar to the Canadian infants at 1 month

**Fig. 2:**
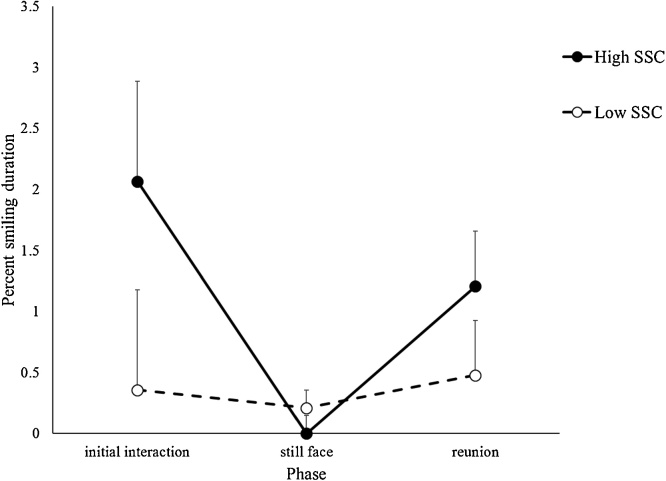
Smiling across the phases of the Still Face Task for Ghanaian infants in the high and low SSC groups. Vertical bars represent standard errors.

Mixed ANOVAs with the repeated variable phase (initial interactive, still face, reunion) and between variable SSC group (high, low) were conducted on the Ghanaian infants’ duration of smiling, non-distress vocalizations, frowning, and distress vocalizations during the Still Face Task. For smiling, there was a significant main effect for phase, *F* (2, 48) = 4.31, *p* =  .019, η_p_^2^ = .152, and significant phase x group interaction, *F* (2, 48) = 3.50, *p* = .038, η_p_^2^ = .127, with no group main effect. Fig. 2 shows the infants’ smiling across the phases for the Ghanaian infants in the high and low SSC groups. The analysis was redone after adjusting for outliers (n = 2 in initial interactive phase, n = 1 in still face phase, n = 2 in reunion phase) by Winsorizing (replacing the outlier’s data with the next closest participant’s data that is not an outlier). The results were unchanged. Ghanaian infants with high SSC experience, but not those with low SSC experience, showed the still face effect with their smiling. There were no significant effects between the Ghanaian infants in the high and low SSC groups for non-distress vocalizations, frowning, or distress vocalizations.

Similar ANOVAs were conducted on the Canadian infants’ duration of smiling, non-distress vocalizations, frowning, and distress vocalizations during the Still Face Task at 1 and 2 months. For smiling, the Canadian infants at 1 month showed a main effect for phase *F* (2, 152) = 3.36, *p* = .037, η_p_^2^ = .042, indicating a linear decline over the phases, with no main effect for group or interaction effect (see Fig. 1b). However at 2 months, the Canadian infants’ smiling showed a significant main effect for phase, *F* (2, 146) = 19.98, *p* < .001, η_p_^2^ = .215, indicating the still face effect, with no group main effect or interaction effect (see Fig. 1b). Thus Canadian infants in both the SSC and control groups did not discriminate between the phases of the Still Face Task with their smiling at 1 month, but infants in both the SSC and control groups did so at 2 months.

However, at 1 month the Canadian infants in the SSC group, but not those in the control group, showed the still face effect with their non-distress vocalizations. A mixed ANOVA yielded a main effect for phase, *F* (2, 152) = 6.86, *p* = .001, η_p_^2^ = .083, and a phase x group interaction, *F* (2, 152) = 3.85, *p* = .023, η_p_^2^ = .048. Infants in the SSC group showed the still face effect with their non-distress vocalizations at 1 month (initial interactive: *M* = 9.9, *SD* = 18.4; still face: *M* = 2.4, *SD* = 3.9; reunion: *M* = 4.6, *SD* = 6.3), whereas infants in the control group did not (initial interactive: *M* = 3.9, *SD* = 4.3; still face: *M* = 2.8, *SD* = 4.3; reunion: *M* = 3.3, *SD* = 4.7). Thus like the Ghanaian infants in the high SSC group, the Canadian infants in the SSC group prior to 2 months of age demonstrated the still face effect with their affect.

The Canadian infants increased their negative affect with their frowns and distress vocalizations through the phases of the task at both 1 and 2 months, as can be seen in Fig. 1d and e. At both ages, infants showed a phase effect for frowns (1 month: *F* (2, 152) = 4.711, *p* = .010, η_p_^2^ = .058; 2 months: *F* (2, 146) = 6.262, *p* = .002, η_p_^2^ = .079) and for distress vocalizations (1 month: *F* (2, 152) = 5.686, *p* = .004, η_p_^2^ = .070; 2 months: *F* (2, 146) = 6.818, *p* = .001, η_p_^2^ = .085), indicating an increase in negative affect from the initial interactive phase to the still face phase that remained or increased at the reunion phase.

### 3.5 Hypothesis 3: Ghanaian infants would show overall behaviors of visual attention, smiling, non-distress vocalizations, frowning, and distress vocalizations that are intermediate between those behaviors found in the Canadian infants at 1 and 2 months

**Fig. 3:**
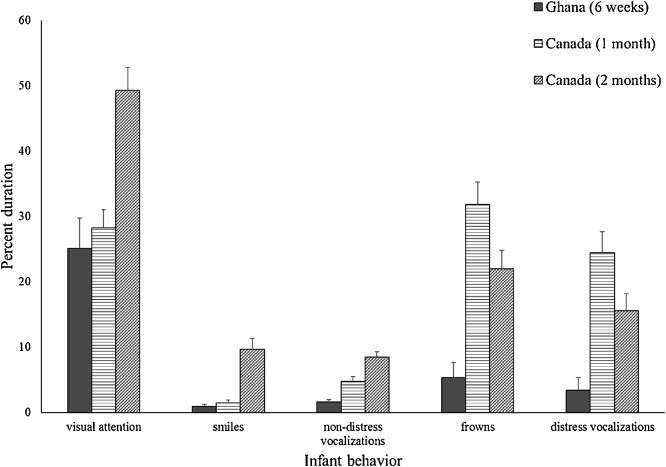
Visual attention, smiling, non-distress vocalizations, frowning, and distress vocalizations during the Still Face Task for Ghanaian infants at 6 weeks and Canadian infants at 1 and 2 months. Vertical bars represent standard errors.

Fig. 3 shows the duration of visual attention, smiling, non-distress vocalizations, frowning, and distress vocalizations during the Still Face Task for the 6-week-old Ghanaian infants and the Canadian infants at 1 month and 2 months. ANOVAs comparing the Ghanaian infants with the Canadian infants at 1 month indicated there was no significant difference in visual attention or smiling between the Ghanaian infants and the Canadian infants, but the Canadian infants were making more non-distress vocalizations, *F* (1, 97) = 5.49, *p* = .021, η_p_^2^ = .054, frowning more, *F* (1, 97) = 19.61, *p* < .001, η_p_^2^ = .168, and making more distress vocalizations, *F* (1, 97) = 14.62, *p* <  .001, η_p_^2^ = .131. ANOVAs comparing the Ghanaian infants with the Canadian infants at 2 months indicated that the Canadian infants were attending more, *F* (1, 99) = 13.87, *p* < .001, η_p_^2^ = .123, smiling more, *F* (1, 99) = 10.25, *p* = .002, η_p_^2^ = .094, making more non-distress vocalizations, *F* (1, 99) = 25.28, *p* < .001, η_p_^2^ = .203, frowning more, *F* (1, 99) = 11.04, *p* = .001, η_p_^2^ = .100, and making more distress vocalizations, *F* (1, 99) = 6.88, *p* = .010, η_p_^2^ = .065. When analyses were redone adjusting for outliers (n = 2 for smiles; n = 1 for non-distress vocalizations; n = 3 for frowns; n = 3 for distress vocalizations) by Winsorizing, the results were unchanged. Thus, rather than showing overall behaviors that were between the amounts shown by the Canadian infants at 1 and 2 months, Ghanaian infants at 6 weeks showed visual attention and smiling behaviors comparable with the Canadian infants at 1 month, but showed less non-distress vocalizations, frowns, and distress vocalizations than Canadian infants at 1 or 2 months.

### 3.6 Hypothesis 4: Ghanaian mothers would show more tactile and less verbal behaviors but similar visual attention and smiling behaviors during the interactive phases of the Still Face Task compared to the Canadian mothers

**Fig. 4:**
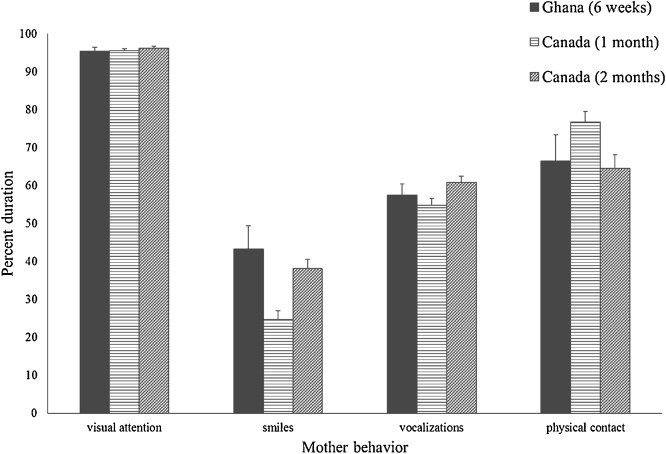
Ghanaian and Canadian mothers’ visual attention, smiling, vocalization, and physical contact with their infants during the interactive phases of the Still Face Task. Vertical bars represent standard errors.

Fig. 4 shows mothers’ duration of visual attention, smiling, vocalizing, and physical contact with their infants during the interactive phases of the Still Face Task for the Ghanaian mothers and for the Canadian mothers at the 1 and 2 month visits. ANOVAs indicated there was no significant difference in the duration of attention, vocalizations, or physical contact with their infants for the Ghanaian mothers and the Canadian mothers at the 1 or 2 month visits. Ghanaian mothers smiled more to their infants than Canadian mothers did at the 1 month visit, *F* (1, 104) = 12.10, *p* < .001, η_p_^2^ = .111, but there was no significant difference in the Ghanaian mothers’ smiling and the Canadian mothers’ smiling at the 2 month visit. Analyses adjusted for outliers (n = 1 for gaze; n = 1 for vocalizations) by Winsorizing did not change the findings.

**Fig. 5:**
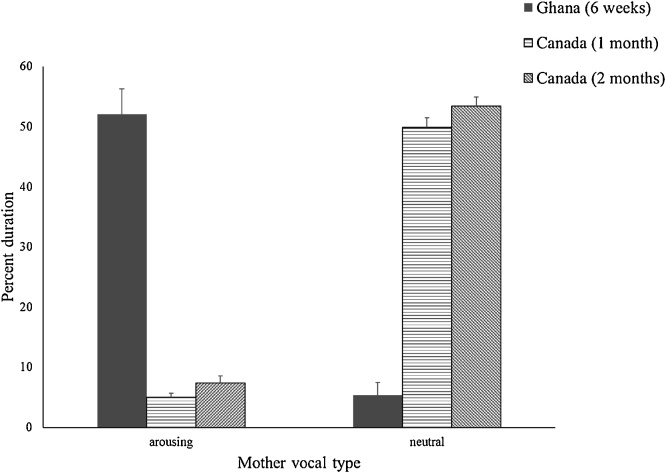
Ghanaian and Canadian mothers’ arousing and neutral vocalizations during the interactive phases of the Still Face Task. Vertical bars represent standard errors.

However, the Ghanaian and Canadian mothers differed on the type of vocalizations and physical contact they did with their infants during the interactive phases of the Still Face Task. Fig. 5 shows the mothers’ vocalizations coded as arousing and neutral. Ghanaian mothers’ vocalizations were more arousing than the Canadian mothers’ vocalizations at the 1 month visit, *F* (1, 97) = 283.62, *p* <  .001, η_p_^2^ = .745, and at the 2 month visit, *F* (1, 99) = 200.87, *p* < .001, η_p_^2^ = .670. Canadian mothers’ vocalizations were more neutral at both the 1 month, *F* (1, 97) = 159.04, *p* <  .001, η_p_^2^ = .621, and 2 month visits, *F* (1, 99) = 184.39, *p* <  .001, η_p_^2^ = .651 than the Ghanaian mothers’ vocalizations. When adjusted for outliers (n = 3 for neutral vocalizations) by Winsorizing, results did not change.

**Fig. 6:**
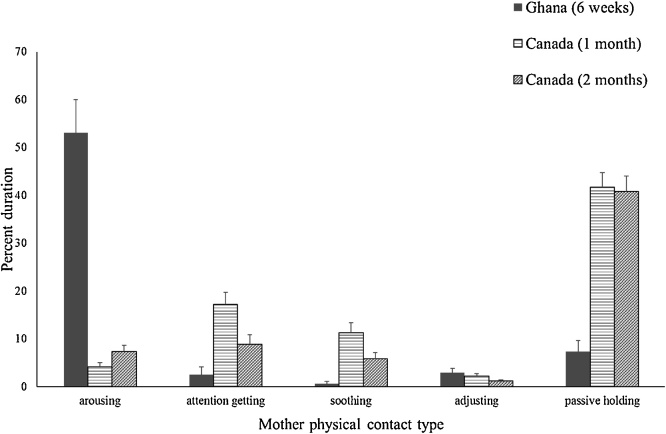
Ghanaian and Canadian mothers’ type of physical contact with their infants during the interactive phases of the Still Face Task. Vertical bars represent standard errors.

Fig. 6 shows the mothers’ physical contact with their infants coded as arousing, attention getting, soothing, adjusting, and passive holding. Ghanaian mothers provided more arousing physical contact with their infants than Canadian mothers at the 1 month visit, *F* (1, 97) = 124.22, *p* <  .001, η_p_^2^ = .562, and at the 2 month visit, *F* (1, 99) = 99.48, *p* < .001, η_p_^2^ = .501. Ghanaian mothers did less attention getting physical contact than Canadian mothers did at the 1 month visit, *F* (1, 97) = 10.95, *p* = .001, η_p_^2^ = .101, but there was no difference at the 2 month visit. Ghanaian mothers did less soothing physical contact than Canadian mothers did at the 1 month visit, *F* (1, 97) = 9.11, p = .003, η_p_^2^ = .086, and at the 2 month visit, *F* (1, 99) = 5.53, *p* =  .021, η_p_^2^ = .053. Ghanaian mothers’ amount of adjusting physical contact was similar to that of Canadian mothers at the 1 month visit, but they did more than Canadian mothers at the 2 month visit, *F* (1, 99) = 7.13, *p* =  .009, η_p_^2^ = .067. Ghanaian mothers did less passive holding than Canadian mothers did at the 1 month visit, *F* (1, 97) = 41.75, *p* < .001, η_p_^2^ = .301, and at the 2 month visit, *F* (1, 99) = 35.21, *p* <  .001, η_p_^2^ = .262. When analyses were redone adjusting for outliers (n = 3 for attention getting; n = 2 for soothing; n = 4 for adjusting; n = 2 for passive holding) by Winsorizing, the only changes in the results were that Ghanaian mothers had less attention getting physical contact than Canadian mothers at the 2 month visit, and Ghanaian mothers and Canadian mothers at the 2 month visit had similar amounts of adjusting physical contact.

As a check to determine whether SSC affected the mothers’ behavior during the Still Face Task, ANOVAs with the between variable group (high SSC, low SSC) were conducted on the Ghanaian mothers’ visual attention, smiling, vocalizations, and physical contact with their infants during the task. There were no significant differences between the groups for any of the behaviors. Likewise, ANOVAs with the between variable group (SSC, control) were conducted on the Canadian mothers’ visual attention, smiling, vocalizations, and physical contact with their infants during the task at the 1 month and 2 month visits. Only the ANOVA for maternal physical contact at the 1 month visit showed a significant group effect, *F* (1, 76) = 6.52, *p* = .013, η_p_^2^ = .079, indicating mothers in the control group provided more physical contact (*M* = 81.8, *SD* = 16.7) during the Still Face Task than mothers in the SSC group (*M* = 67.3, *SD* = 31.4). Follow-up ANOVAs were conducted on the type of physical contact mothers provided for their infants at the 1 month visit. Control group mothers provided more passive holding (*F* (1, 76) = 6.09, *p* = .016, η_p_^2^ = .074; SSC group *M* = 31.8, *SD* = 28.0; control group *M* = 47.0, *SD* = 24.6) and SSC group mothers provided more soothing physical contact (*F* (1, 76) = 4.51, *p* = .037, η_p_^2^ = .056; SSC group *M* = 17.2, *SD* = 24.7; control group *M* = 8.1, *SD* = 13.5).

Ghanaian mothers and Canadian mothers were similar in the amount of attention, smiling, vocalizing, and physical contact directed toward their infants during the interactive phases of the Still Face Task, with the exception that Ghanaian mothers smiled more than Canadian mothers at the 1 month visit. However, Ghanaian mothers were more arousing in their vocalizations and physical contact with their infants than Canadian mothers. Canadian mothers’ vocalizations were more neutral and their physical contact with their infants was used for more attention getting, soothing, and passive holding. Adjusting physical contact was minimal for both groups.

## 4 Discussion

Ghanaian mother-infant dyads at the infant age of 6 weeks showed both similarities and differences in their interactions in the Still Face Task compared to Canadian mother-infant dyads at the infant ages of 1 and 2 months. Importantly, Ghanaian infants with high SSC experience, but not those with low SSC experience, showed affect responses to their mothers’ changing behavior during the task in ways that were similar to Canadian infants in the SSC group at 1 month.

Ghanaian infants in both the high and low SSC groups showed the still face effect with their visual attention, providing support for the first hypothesis. The Canadian infants at 1 and 2 months also showed the still face effect with their visual attention. Young infants from both cultural contexts demonstrated with their attention that they detected changes in the mothers’ behavior. Indeed, Bigelow and Power (2012), who longitudinally followed the Canadian infants from 1 week to 3 months of age, found that even at 1 week the infants showed the still face effect with their attention. Although few other studies have used the Still Face Task with newborns, the ones that have done so indicate that infants reduce their attention from the initial interactive phase to the still face phase (Bertin & Striano, 2006; Ellsworth, 1987, cited in Muir & Hains, 1993; Nagy, 2008). Thus infants show evidence of detecting changes in their mothers’ interactive behavior from the beginning of life.

Ghanaian infants with high SSC experience, but not those with low SSC experience, showed the still face effect with their affect. They smiled more in the interactive phases than the still face phase, providing support for the second hypothesis. Researchers speculate that the systems that regulate infants’ attention and affect operate differently during face-to-face interactions (Bigelow & Walden, 2009; Legerstee & Varghese, 2001; Nadel, Soussignan, Canet, Libert, & Gerardin, 2005). They propose that changes in infants’ visual attention during the Still Face Task indicate infants’ detection of changes in their mothers’ behavior, whereas changes in infants’ affect indicate they are responding to violations of their expectations for affect sharing, which develops later than infants’ ability to detect changes in their mothers’ behavior. Ghanaian infants with high amounts of SSC affectively responded to their mothers’ changing behavior during the Still Face Task, suggesting SSC accelerated the infants’ expectations for affect sharing.

For Canadian infants, there was no evidence of the still face effect for smiling at 1 month, however by 2 months, infants in both the SSC and control groups showed the still face effect with their smiling, which is evidence of the 2 month transition when infants become more interested and participatory social partners (Henning et al., 2005; Lavelli and Fogel, 2002, Lavelli and Fogel, 2005; Rochat, 2001). Yet Canadian infants in the SSC group at 1 month showed the still face effect with their affect via their non-distress vocalizations. Although smiling is the most frequently used measure of infants’ positive affect, infants’ non-distress vocalizations convey their emotional reactions and are the primary social signals that elicit maternal communication (Hsu & Fogel, 2001; Keller, Lohaus, Volker, Cappenberg, & Chasiotis, 1999; Papousek, 1989; Van Egeren, Barratt, & Roach, 2001). Mothers use these vocalizations as signals for determining infants’ readiness to interact and for adjusting their own emotional response. Thus both Ghanaian and Canadian infants with high SSC experience responded to the Still Face Task with their affect prior to 2 months of age.

Why would Ghanaian infants’ early affect response to the Still Face Task be with smiling and Canadian infants’ early affect response be with non-distress vocalizations? Kärtner et al., 2008, Kärtner et al., 2010 found that the Nso mothers of the Cameroon were as contingently responsive to their infants as German mothers, but their predominant mode of responsiveness was expressed through physical contact. German mothers responded contingently to their infants primarily visually with gaze, smiles, and facial expressions and with verbal turn-taking. Nso mother-infant dyads engaged less in face-to-face interactions with periods of mutual gaze. Thus Nso infants experienced their mothers’ smiling during maternal interactions less often. Yet when Nso mothers were in mutual gaze with their infants, they smiled as much as German mothers. The Still Face Task involves mother-infant face-to-face interaction with mutual gaze. Sroufe (1996) proposed that the emergence of social smiling is facilitated by infants’ recognition of others’ contingent responsiveness to their behavior, not only in face-to-face contexts but also in contexts of other modalities, such as body contact or stimulation. SSC has been shown to facilitate mothers’ responsiveness to their infants’ signals as well as infants’ sensitivity to their mothers’ behavior toward them (Feldman, 2004; Feldman, Eidelman, et al., 2002). Such increases in mother-infant reciprocity may explain the Ghanaian infants in the high SSC group being more reactive with their smiling to their mothers’ changing behavior during the task. Canadian infants would have more face-to face experience with their mothers in which verbal turn-taking is prevalent. Although mothers also smile responsively in face-to-face interactions with their infants, mothers’ vocal responsiveness may be more easily noticed. Young infants’ smiling is instigated by maternal smiling (Kaye & Fogel, 1980), therefore infants’ smiles tend to overlap with mothers’ smiles. In Western societies where mother-infant vocal turn-taking is prevalent, when infants vocalize, overlap with maternal talking is minimal (Bigelow, MacLean et al., 2010; Bornstein et al., 1992). Mothers tend to stop talking and resume after infant vocalizations have ended, resulting in vocal turn-taking. Infants who experience such turn-taking may recognize their mothers’ responsiveness more readily with vocalizations than with smiles.

Interestingly, SSC experience affected infants’ behavior toward their mothers in the Still Face Task, yet SSC did not influence the behavior of the mothers toward their infants during the task. Ghanaian mothers in both high and low SSC groups showed similar behavior toward their infants. Canadian mothers in SSC and control groups also showed similar behavior toward their infants, with the exception that mothers in SSC and control groups differed in the amount of physical contact with their infants during the Still Face Task at 1 month. Mesman et al. (2009) indicate that the history of infants’ experience with their mothers is an important aspect affecting infants’ response to the Still Face Task. The findings suggest that the infants’ history of SSC experience with their mothers was a significant factor influencing the infants’ response to the task.

Ghanaian infants’ overall duration of attention and smiling during the Still Face Task paralleled the overall duration of those behaviors by the Canadian infants at 1 month, rather than at 2 months. Kärtner et al. (2010) found that, although Nso infants experience less mutual gaze with their mothers, when in face-to-face interaction with their mothers, Nso infants at 6 weeks gazed and smiled at their mothers as much as German infants at this age (Wörmann et al., 2012). The Ghanaian infants, however, engaged in less non-distress vocalizing than the Canadian infants at 1 month, possibly because the Canadian infants in the SSC group at 1 month were already responding to the Still Face Task with their non-distress vocalizations. By 2 months, the Canadian infants engaged in more attention, smiling, and non-distress vocalizing than the Ghanaian infants. In comparing German and Nso mothers’ interactions, cultural differences in the modes of maternal responsiveness emerged in the infants’ second and third months (Kärtner et al., 2010). The divergence was mostly due to German mothers decreasing their tactile responsiveness and increasing their visual responses of gaze, smiles, and facial expressions; whereas Nso mothers maintained their high level of tactile responsiveness with little increase in visual responses. Mother-infant interactions are transactional in that both partners influence each other. Yet mothers are primarily responsible for establishing and maintaining interactions with their infants, particularly in the infants’ early life (Henning & Striano, 2011; Kaye & Fogel, 1980). Ghanaian infants’ experience of maternal responsiveness may be more similar to that of Canadian infants at 1 month, prior to the divergence of cultural modes of maternal responsiveness, than that of Canadian infants at 2 months.

Notably, Ghanaian infants differed from Canadian infants at both 1 and 2 months in distress, as expressed in frowns and distress vocalizations of fussiness and crying. Ghanaian infants were less distressed during the Still Face Task. Proximal parenting cultures value calmness in infants and the suppression of negative affect (Keller & Otto, 2009). Mothers in both Western and non-Western cultures tend to respond readily to infant distress (Mesman et al., 2018). Yet the physical closeness of Ghanaian mother-infant dyads, through mothers carrying their infants and sleeping with them, facilitates the mothers’ prompt response to, and anticipation of, infant distress, which promotes mothers’ nurturance of calmness in their infants.

Ghanaian and Canadian mothers were similar in the amount of infant directed behaviors they provided during the interactive phases of the Still Face Task. Nso mothers were found to smile as much as German mothers when in mutual gaze with their infants, despite mutual gaze being less frequent for Nso mother-infant dyads (Kärtner et al., 2010; Wörmann et al., 2012). The Still Face Task involves face-to-face interaction in which mutual mother-infant gaze is prevalent. In this context, the amount of maternal attention, smiling, vocalizing, and physical contact was similar for Ghanaian and Canadian mothers, even though Ghanaian mothers smiled more than Canadian mothers when their infants were 1 month. However, the mothers’ type of vocalizations and physical contact with their infants differed. Ghanaian mothers’ vocal and physical contact behaviors were more arousing. Their vocalizations tended to be boisterous rhythmic sounds, energetic and repetitive infant greetings and singing. These vocal behaviors were accompanied by moving the infants’ limbs in rhythm with the vocalizations. Maternal touch is important in regulating infant emotion. In Western societies, mothers use touch to reduce distress and elicit positive affect (Stack & Arnold, 1998; Stack & Muir, 1992). Canadian mothers’ tactile behavior was most often used for soothing the infant, getting the infant’s attention, or simply passively touching the infant, and their vocalizations were less stimulating.

Mothers from proximal caretaking cultures of West Africa have been described as treating their infants as novices who need to learn compliance and subordination (Kärtner et al., 2010). Mothers are directive in this process, which influences the ways in which they engage with their infants. Mothers tend to lead the interaction rather than follow the infant; synchrony rather than reciprocity is prominent, which promotes the cultural goals of interdependency and relatedness (Keller et al., 2004). Mother-infant interaction is characterized by rhythmic structuring with musical chorusing and repeated greetings, synchronized with rhythmic body stimulation. These modes of interaction were seen in the Ghanaian mothers’ behaviors in the interactive phases of the Still Face Task. Ghanaian infants were primarily calmly attentive to these energetic behaviors of their mothers.

There are several limitations to the study. The Ghanaian mothers, coming from a proximal parenting culture, were less accustomed to face-to-face interactions with their infants (Kärtner et al., 2008; Keller, 2007; Keller et al., 2004), which may have affected the way in which they vocally and tactually engaged with their infants during the interactive phases of the Still Face Task. Yet the Ghanaian mothers’ behavior was similar to that described by other researchers studying mother-infant engagement during naturalistic face-to-face interactions in West African cultures (Kärtner et al., 2010). Although the study examined Ghanaian mothers’ behaviors toward their infants during the Still Face Task, the study did not assess the mothers’ contingent responsiveness to their infants’ behavior. Mothers from West African societies tend to be predominantly tactually responsive to their infants, but not necessarily while in mutual gaze with their infants (Kärtner et al., 2008, Kärtner et al., 2010). Thus, to assess Ghanaian mothers’ contingent responsiveness would require measuring their tactile responsiveness during naturalistic mother-infant interactions, which is beyond the scope of the current data. The amount of daily SSC Ghanaian and Canadian mothers provided for their infants during the infants’ first month was based on mothers’ self reports. It is possible that the mothers did not accurately report the amount. The sample size of Ghanaian mother-infant dyads was small, which reduces the power of analyses, particularly in finding significant results. That significant results were obtained with the limited sample size suggest the findings are robust. Yet the size of the sample limits the interpretation of the results. The Ghanaian mother-infant dyads were seen in a cross-sectional study at the infant age of 6 weeks, whereas the Canadian archival data to which they were compared come from a longitudinal study at the infant ages of 1 and 2 months. Both studies examined the effect of SSC on infants’ response to the Still Face Task prior to the 2 month transition; and the Canadian study is the only known SSC study utilizing the Still Face Task in Western culture with infants at ages comparable to the Ghanaian infants. Nevertheless, the differences in the study designs may have affected the results found between the two cultures. The length of the initial interaction differed slightly in the Ghanaian and Canadian studies (2 min and 3 min, respectively), although length of episodes has been shown to have little effect on infants’ response to the task (Adamson & Frick, 2003; Mesman et al., 2009). The infants were seated in a car seat for the Still Face Task, which may have been novel for some of the Ghanaian infants as the use of car seats is not necessarily common practice in Ghana. Yet the Ghanaian infants did not resist the seat and did not seem distracted by it. Ghanaian mothers’ demographics differed from those of the Canadian mothers, which may have affected their maternal behaviors. However, the demographics of the Ghanaian mothers are prototypical of their cultural context, and thus are interwoven with their parenting goals and practices.

Despite cultural differences in how mothers engaged with their infants, mother-infant SSC facilitated infants’ early expectations for their mothers’ behavior. Ghanaian infants with high SSC experience, on the cusp of the 2 month transition, responded with their smiling to changes in their mothers’ behavior during the Still Face Task, just as Canadian infants in the SSC group prior to 2 months responded with their affect to their mothers’ behavioral changes during the task. In both cultures, SSC enhanced infants’ ability to emotionally respond to their mothers’ social behavior, suggesting acceleration of the infants’ developing expectations for their mothers’ engagement.

## Funding

This research was aided by a grant from the Bill and Melinda Gates Foundation to the first (Principal Investigator) and second authors and by a grant from the Nova Scotia Health Research Foundation to the second author. The funders had no involvement in the design, conduction, or writing of the report. Gratitude is expressed to Yasmin Mohammed, Jan Hanifen, Gerry Cameron, Rachel MacFarlane, Mena Enxuga, Jennifer Delaney, Yvonne MacDonald, Cynthia Flannigan, Lynne Lukeman, Dale Fewer, Charlene Kennedy-Chisholm, Chow Shim Pang, Laura Walden, Caitlin Best, and Madison Links, who were research assistants; and to the mothers and infants who participated in the study.

## Declaration of Competing Interest

None.
